# The tissue specific regulation of miR22 expression in the lung and brain by ribosomal protein L29

**DOI:** 10.1038/s41598-020-73281-z

**Published:** 2020-10-01

**Authors:** Mohammad Ishaque Ali, Linrui Li, Lexing Li, Lun Yao, Jie Liu, Wei Gu, Shuguang Huang, Bingyu Wang, Guoquan Liu

**Affiliations:** 1 Department of Biochemistry and Molecular Biology, School of Medical Laboratory, Anhui Province Key Laboratory of Translational Cancer Research, Bengbu Medical College, 2600 Donghai Street, Bengbu, 233030 Anhui People’s Republic of China; 2 Department of Basic Veterinary Medicine, College of Veterinary Medicine, Huazhong Agricultural University, Wuhan, 430070 Hubei People’s Republic of China; 3 Department of Livestock Services, Dhaka, People’s Republic of Bangladesh

**Keywords:** Biochemistry, Cell biology, Molecular biology

## Abstract

Endogenous miR22 is associated with a diverse range of biological processes through post-translational modification of gene expression and its deregulation results in various diseases including cancer. Its expression is usually tissue or cell-specific, however, the reasons behind this tissue or cell specificity are not clearly outlined till-date. Therefore, our keen interest was to investigate the mechanisms of tissue or cell-specific expression of miR22. In the current study, miR22 expression showed a tissues-specific difference in the poly(I:C) induced inflammatory mouse lung and brain tissues. The cell-specific different expression of miR22 was also observed in inflammatory glial cells and endothelial cells. The pattern of RPL29 expression was also similar to miR22 in these tissues and cells under the same treatment. Interestingly, the knockdown of RPL29 exerted an inhibitory effect on miR22 and its known transcription factors including Fos-B and c-Fos. Fos-B and c-Fos were also differentially expressed in the two cell lines transfected with poly(I:C). The knockdown of c-Fos also exerted its negative effects on miR22 expression in both cells. These findings suggest that RPL29 might have regulatory roles on tissue or cell-specific expression of miR22 through the transcription activities of c-Fos and also possibly through Fos-B.

## Introduction

MicroRNAs (miRNAs) are non-coding RNAs, usually contains about 22–25 nucleotides and found abundantly in cells and extracellular environment of an organism in animal and plants kingdom. These are evolutionary conserved and control gene action or expression either by stimulating transcription or degradation and translational repression of various target specific mRNA by binding to the 3′untranslated region^1,2^. MiRNA-22-3p (miR22) is a 22-nucleotide microRNA and primarily discovered in HeLa cells. It is an exonic miRNA, located on the 2nd exon of gene MGC14376^3^. Numerous studies indicate that miR22 is a vital player in various pathophysiological functions including metabolism, hematopoiesis, cell division, growth, adhesion, senescence, apoptosis, angiogenesis, fibrogenesis, tissue homeostasis, tissue remodeling and inflammation^4–9^. Recent numerous studies have established the fact that the miR22 plays active roles in the immune cascades of an organism and its dysregulation is associated with different inflammatory diseases, including atopic dermatitis, asthma, psoriasis, rheumatoid arthritis, inflammatory bowel disease, atherosclerosis, multiple sclerosis, emphysema and myocardial or cerebral ischemia–reperfusion injury^10–15^*.* MiR22 plays critical roles in different cancers involving different pathophysiological processes via activation or deactivation of various target genes or mRNAs associated with many signaling cascades^7,9,16,17^. It acts as either oncogene or suppressor, serves as a marker for diagnosis and surveillance, or as a sensitizer in the treatment of various cancer^18^. MiR22 is also intimately involved in different cardiovascular diseases and is associated with diabetes and neural diseases like Parkinson’s^8,19–22^.

MiR22 is usually available in various tissue, but the expression is relatively higher in heart, smooth muscle, adipose tissue, and bladder^23^. It is reported that miR22 is upregulated in the serum of both hepatitis B&C virus-infected patient but downregulated in hepatitis B virus-related hepatocellular carcinoma cell lines and clinical tissues^24,25^. Scientists also reported that expression of miR22 could be modified by the various stimulating agents such as Phorbol-12-myristate-13-acetate, polyinosinic-polycytidylic acid (poly(I:C)), endosulfan, IL-1α, extracellular ATP/UTP and upon several viral infections^5,16,26–28^. The poly(I:C) is a synthetic analog of viral dsRNA analog and have been used as viral mimic or an immune stimulant in various in vitro or in vivo studies. Interestingly, the miR22 is upregulated in glial cells but downregulated in endothelial cells in response to poly(I:C) and plays differential role in inflammation targeting MAVS (mitochondrial antiviral signaling) and VE-cadherin in these two cell lines respectively^5,28^. Many other studies also confirmed the tissue or cell specific expression of miR22^23^.

Ribosome protein L29 (RPL29) is a structural component of ribosomal 60 s subunit of the cell and plays critical roles in diverse biological processes such as cell cycle control, cell proliferation, cell differentiation and angiogenesis^29,30^. Our previous RNA-seq study showed the different expression of RPL29 in inflammatory mouse brain and lung tissues after poly(I:C) treatment^31^. This led to our hypothesis that there might be regulatory inter-relationship between RPL29 and miR22 or RPL29 might be responsible for the tissues specificity of miR22. In the present study, we investigate the pattern of RPL29 and miR22 expression in endothelial and glial cells and in the mouse lung and brain tissues in response to poly(I:C) treatment to outline the inter-relationship of these two molecules and the mechanisms of different and tissue specific expression of miR22. The findings of our study might provide insight clue for the miR22 regulation.

## Results

### The expression of miR22 is different depending on the cell or tissue

**Figure 1:**
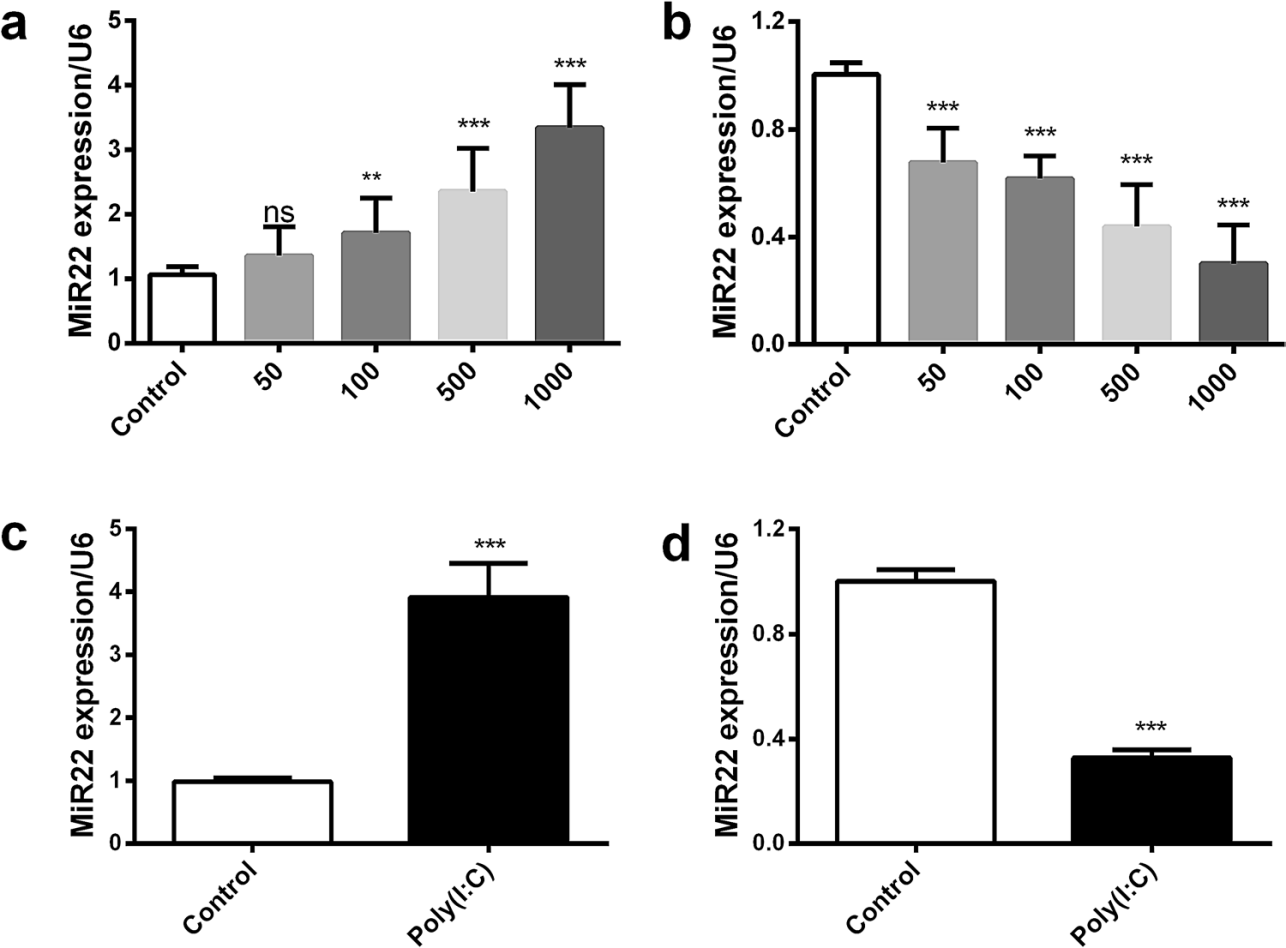
The expression of miR22 in different cells and mouse tissues in response to poly(I:C). Human glioblastoma astrocytoma cells (U251) and human umbilical vein cells (EA.hy926) were transfected with poly(I:C) at the different concentrations for 6 h. The miR22 level in U251 cells (**a**) and EA.hy926 cells (**b**) was determined with qRT-PCR using U6 as an internal control. Mice were treated with 100 µg of poly(I:C) for 24 h and miR22 level of the brain (**c**) and the lung (**d**) tissues were quantified by qPCR. All qPCR data are representative of three independent experiments with three replicates. (**P < 0.01; ***P < 0.001).

To observe the pattern of miR22 expression in different cells, we treated human glial cells (U251) and human endothelial cells (EA.hy926) with poly(I:C) at different concentration. The expression of miR22 was detected through qPCR. The miR22 was upregulated in U251 and downregulated in EA.hy926 cells in a dose depended manner (Fig. 1a,b). Next, to investigate its expression in different tissues, we quantified the miR22 in the lung and brain tissues of mouse after poly(I:C) treatment through intra-peritoneal injection. The miR22 was upregulated in the brain but downregulated in the lung tissues (Fig. 1c,d). These results indicated that the expression of miR22 is cell and tissue-specifically regulated.

### MiR22 and RPL29 are positively correlated in two different tissues and cell lines

**Figure 2:**
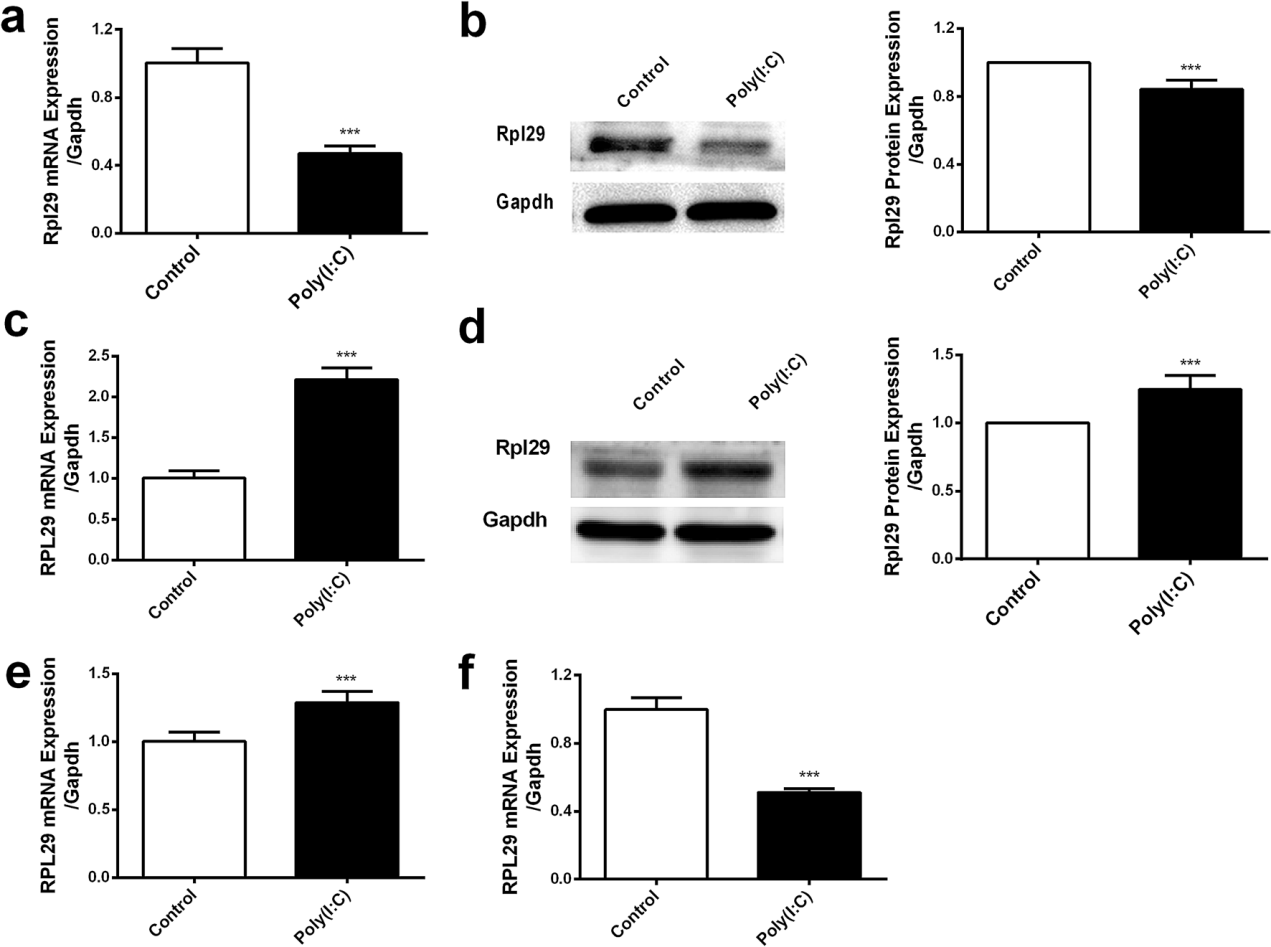
RPL29 expression in the brain and lung tissues of mouse and in two different cell lines in response to poly(I:C). RPL29 mRNA was quantified in the lung (**a**) and brain (**c**) tissues of mice treated with poly(I:C) for 24 h through qPCR. RPL29 protein was quantified in the lung (**b**) and brain (**d**) tissues of mice with similar treatment through western blot. Full-length blots/gels are presented in Supplementary Figure S2-S5. RPL29 mRNA was also detected in poly(I:C) treated U251 (**e**) and EA.hy926 (**f**) cells through qPCR. All data were curated from three independent experiment with three replication and represented as mean ± SD (***P < 0.001).

Our previous study confirmed the upregulation of RPL29 in the inflammatory brain and its downregulation in the inflammatory lung tissues of poly(I:C) treated mice through RNA-seq^31^. We further investigate the expression of RPL29 at mRNA and protein levels in these two tissues with similar treatment through qPCR and Western blotting. The RPL29 mRNA and protein were downregulated in the lung (Fig. 2a,b, Supplementary Figure S1a) but upregulated in the brain (Fig. 2c,d, Supplementary Figure S1b) tissues. The expression of this gene was also investigated in poly(I:C) treated EA.hy926 and U251 cell lines. We treated these cell lines with poly(I:C) at the concentration of 100 ng/ml for 6 h. The miR22 expression was different in U251 and EA.hy926 cells under this treatment. The expression of *RPL29* was detected through qRT-PCR. As expected, the RPL29 mRNA was upregulated in U251 cells (Fig. 2e) and downregulated in EA.hy926 cells (Fig. 2f). These results highlighted the positive correlation between RPL29 and miR22 expression during inflammation.

### RPL29 plays regulatory roles on the expression of miR22

**Figure 3:**
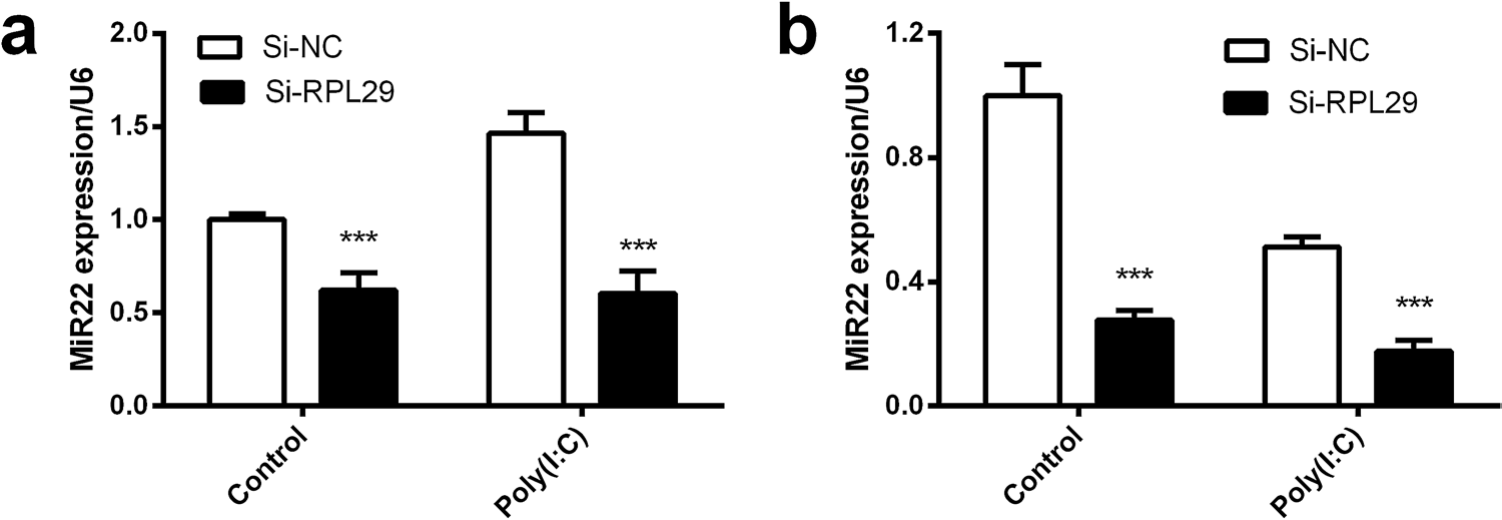
The expression of miR22 in RPL29 knocked-down cells. Human glioblastoma astrocytoma cells (U251) and human umbilical vein cells (EA.hy926) were transfected with si-RPL29 for 24 h followed by poly(I:C) treatment at the concentration of 100 ng/ml for 6 h. The expression miR22 in U251 cells (**a**) and EA.hy926 cells (**b**) was determined with qRT-PCR using U6 as an internal control. All data are representative of at least three independent experiments with three replicates. (***P < 0.001).

The positive correlation between the RPL29 and miR22 expression in the poly(I:C) treated mouse brain and lung tissues, and also in two different cell lines, indicating that there might have a regulatory interaction among them. Firstly, we hypothesized that miR22 might be a regulator of RPL29 expression. We searched for transcription factors of RPL29 and target genes of miR22 in different online databases. However, RPL29 is not the target gene of miR22 and miR22 is not transcription factors of RPL29. Therefore, we thought that RPL29 might be an upstream gene and one of the regulator of miR22. To confirm this idea, we knocked-down RPL29 with siRNA in both U251 and EA.hy926 cells for 24 h followed by poly(I:C) treatment for further 6 h and quantified miR22 through qRT-PCR. We found that RPL29 knockdown decreased the poly(I:C) stimulated miR22 in U251 cells (Fig. 3a) and showed an additive effect of poly(I:C) induced downregulation of miR22 in EA.hy926 cells (Fig. 3b). These results indicated that the RPL29 might have a role in regulation of miR22 expression.

### RPL29 regulates miR22 expression via regulating Fos-B and c-Fos expression

**Figure 4:**
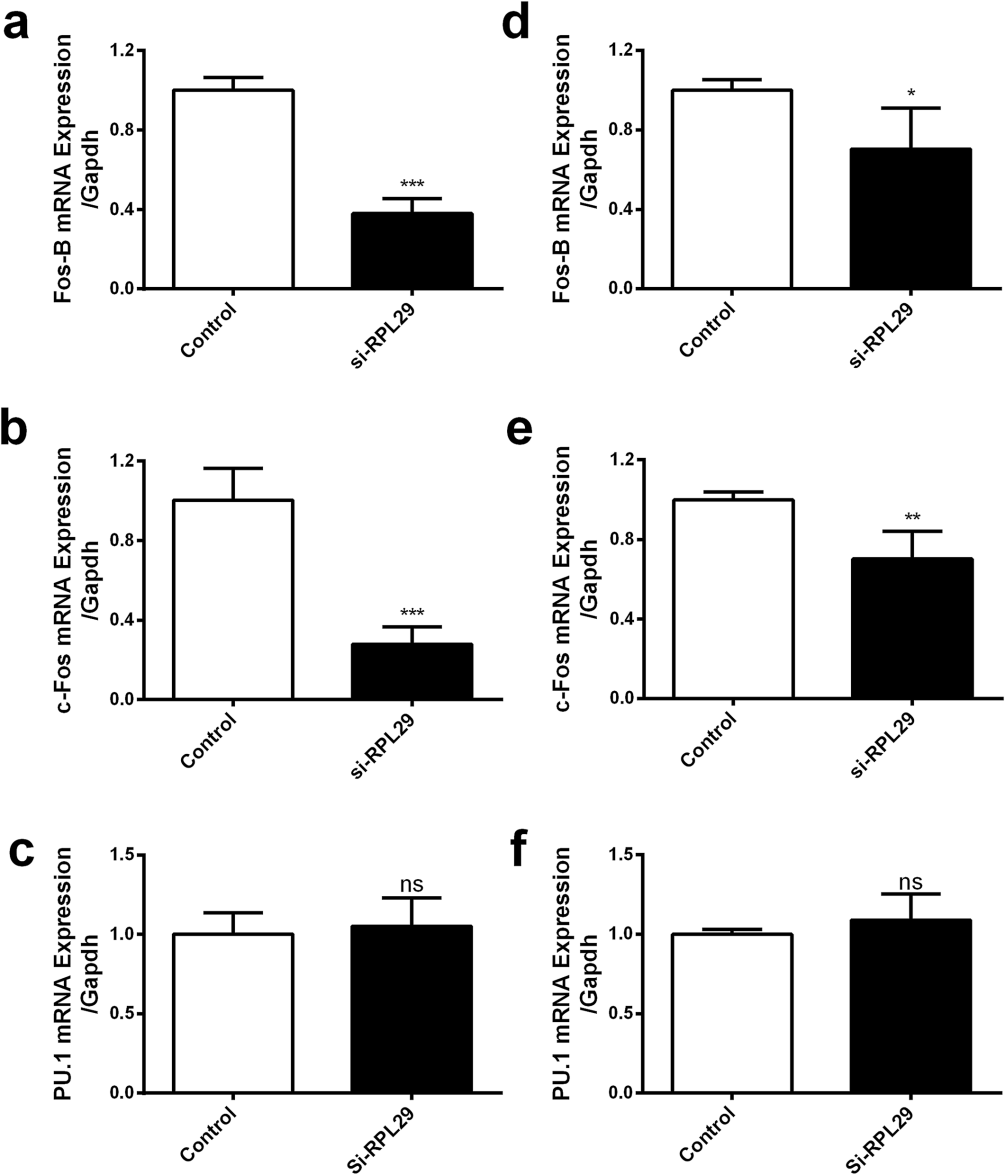
Expression of transcription factors of miR22 in RPL29 knocked down cells. U251 and EA.hy926 cells were treated with RPL29 si-RNA for 24 h at the final concentration of 50 nM, and the mRNA expression of Fos-B, c-Fos, and PU.1 in U251 (**a**–**c**) and EA.hy926 (**d**–**f**) cells were determined respectively through qRT-PCR. GAPDH was used as an internal control, and all data were curated from three independent experiments having three replicates. (*P < 0.05; **P < 0.01; ***P < 0.001).

**Figure 5:**
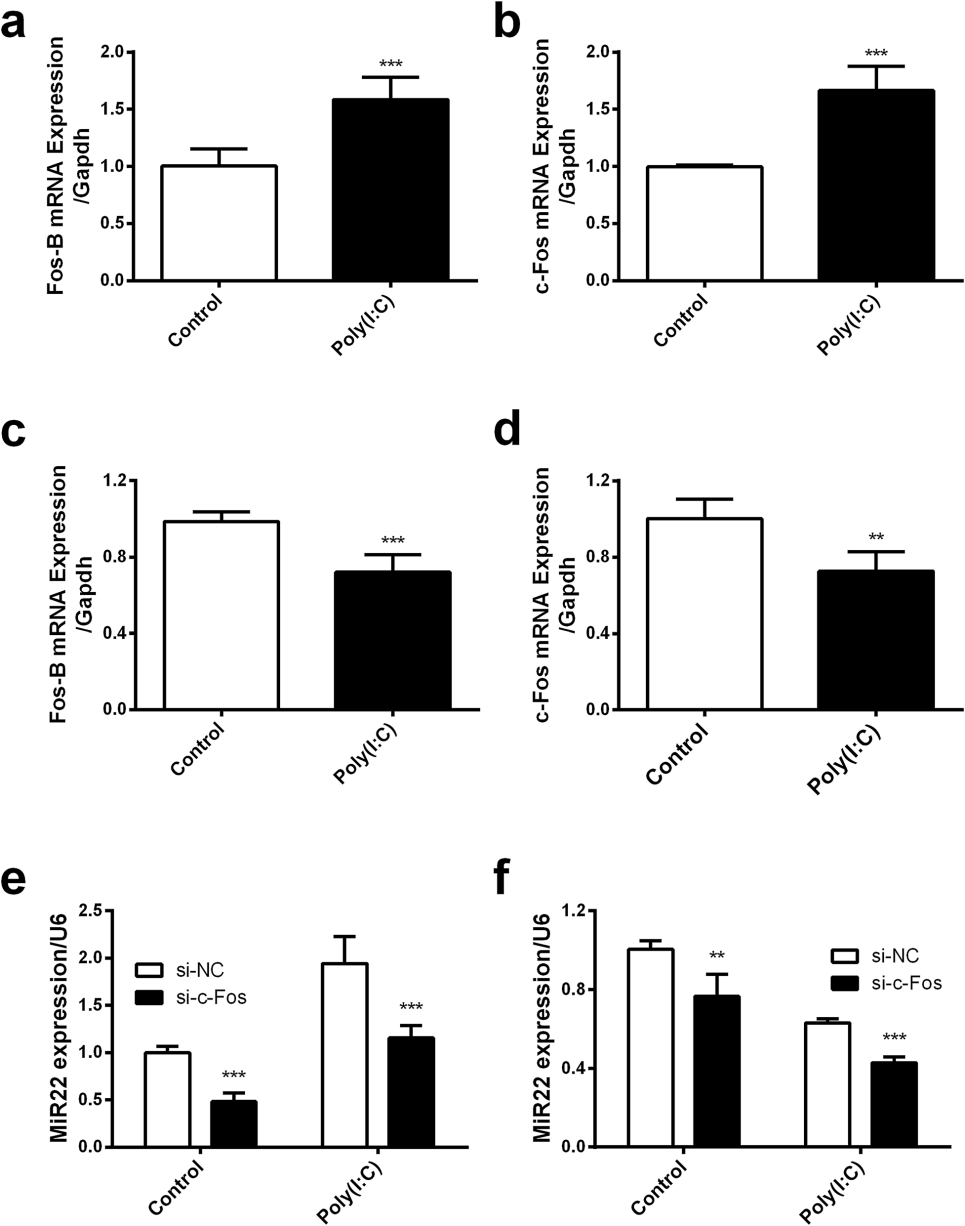
The expression of Fos-B and c-Fos in different cells upon poly(I:C) treatment and the expression of miR22 in c-Fos knocked down cells. Fos-B and c-Fos mRNA were measured in U251 (**a**–**b**) and EA.hy926 (**c**–**d**) cells transfected with poly(I:C) for 6 h through qRT-PCR. Later on c-Fos was knocked down with si-RNA (Final concentration 50 nM) in both types of cells and transfected with poly(I:C) (100 ng/ml) for another 6 h. The expression of miR22 was quantified in U251 (**e**) and EA.hy926 (**f**) cells. All data were curated from three independent experiment with three replications (*P < 0.05; **P < 0.01; ***P < 0.001).

MiR22 can regulate the expression of numerous genes post-transcriptionally. Besides, many transcription factors are responsible for the regulation of miR22 expression. Some studies confirmed the genes FosB, c-Fos, PU.1, P53, NFκB, and AKT as the transcription regulators of miR22 in different cells^10,15,27,32–34^. However, RPL29 is not a transcription factor of miR22. Therefore, to find out the mechanism of miR22 regulation by RPL29, we selected transcription factors FosB, c-Fos, and PU.1, for experimentally validation, and detected their expression in the siRNA mediated RPL29 inhibited cells. The Fos-B and c-Fos were significantly downregulated; however, PU.1 did not respond significantly (Fig. 4a–f). We also investigated the effects of poly(I:C) on the expression of these two significant transcription factors in U251 and EA.hy926 cell lines. It was found that both Fos-B and c-Fos were upregulated in U251 (Fig. 5a,b) but downregulated in EA.hy926 cells (Fig. 5c,d). Furthermore, to confirm the regulatory roles of these two transcription factors for miR22, we treated these two experimental cell lines with c-Fos siRNA and quantified the miR22 expression. The results showed that knockdown of c-Fos exerts its down regulatory effect on poly(I:C) stimulated upregulation of miR22 in U251 cells (Fig. 5e). Whereas c-Fos knock-down had an additive effect on poly(I:C) induced downregulation of miR22 in EA.hy926 cells (Fig. 5f). These results indicated that RPL29 might regulate the expression of miR22 through the transcription activities of c-Fos, and possibly through that of FosB.

## Discussion

MiRNA is a new dimension of gene regulator and associated with various biological processes of an organism. MiR22 is involved in different pathophysiological mechanisms including cell division, cell cycle, angiogenesis, inflammation and immunity via controlling the expression of its target genes. The regulation of miR22 is a critical issue to the scientists for its Janus-faced nature in inflammation and cancer. In the current study, the expression of miR22 in the viral mimic-induced inflammatory mouse lung and brain tissues was different. MiR22 was upregulated in the brain and downregulated in the lung. The expression pattern of miR22 was also different in U251 and EA.hy926 cell lines upon poly(I:C) treatment. These studies confirmed that the expression of this miRNA in response to viral mimic is cells and tissue-specific.

It is reported that miR22 is upregulated in the serum of both hepatitis B virus and hepatitis C virus-infected patient, but downregulated in hepatitis B virus-related hepatocellular carcinoma cell lines and clinical tissues^24,25^. It is showed that miR22 is upregulated in PRRSV infected porcine lung of Tongcheng breed but not in the lung of Landrace breed under the same condition^35^. Some scientists also reported that miR22 is differentially expressed in the asthmatic and non-asthmatic pBEC cell upon influenza A (H1N1) virus infection^36^. Differential expression of miR22 can regulate the transmissible gastroenteritis virus-induced inflammation in intestinal porcine epithelial cell-jejunum 2 cell line^37^. The upregulation of miR22 regulates inflammatory responses in influenza A virus-infected cells and promotes viral replication through targeting hosts *HO-1* gene expression in PRRSV infected mouse lung^35,36,38^. The higher expression of miR22 in heart, smooth muscle, adipose tissue, and bladder is reported besides its ubiquitously expression in various tissues^23^. All the findings are agreed our results of tissue specificity of miR22 expression. In addition, miR22 regulates inflammation or immune responses either positively or negatively in different cells and tissues under various diseased conditions^5,10,26,28,39,40^. Therefore, we sought to outline the mechanisms of cell or tissue specific expression of miR22.

RPL29 expression was also different and showed similar pattern like miR22 in the inflammatory brain and the lung tissues of the mouse as well as in EA.hy926 and U251 cell lines after poly(I:C) treatment. These results reflected a co-expression or a probable regulatory inter-relationship between miR22 and RPL29 in theses tissues and cells. Some transcription factors can regulates the expression of miR22 and miR22 contributes to post-translational regulation of many genes as its targets. RPL29 is neither the target gene nor the transcription factor of miR22. However, knockdown of RPL29 suppresses poly(I:C) triggered miR22 in U251 cells and had an additive effect on poly(I:C) mediated suppression of miR22 in EA.hy926 cells. Besides these, RPL29 knockdown results in the down expression of Fos-B and c-Fos in these two cell lines. The Fos-B and c-Fos are two experimentally validated transcription factors of miR22^27,32^. These findings suggested that the RPL29 might regulate the expression of miR22 through the transcriptional activities of the AP-1 protein family members Fos-B and c-Fos. Poly(I:C) treatment also results in Fos-B and c-Fos upregulation in U251 and downregulation in EA.hy926 cells. c-Fos knockdown results in downregulation of poly(I:C) modulated miR22 in both U251 and EA.hy926 cells. Collectively these results indicated that the poly(I:C) induced differential expression of miR22 in the mouse lung and brain tissues and in the glial and endothelial cells might be regulated by RPL29. Recent numerous studies confirmed that the expression of miR22 is regulated transcriptionally along with its host gene by some transcription factors including NF-κB, p53, Fos-B, c-Fos, PU.1, AKT Jak3, STAT3, and STAT5^10,15,27,32–34,41^. There are some cellular and extracellular stimuli including polyinosinic-polycytidylic acid (poly(I:C)), phorbol-12-myristate-13-acetate, endosulfan, IL1α, nicotinamide phosphoribosyltransferase, 12-o-tetradecanoylphorbol-13-acetate, and xendin-4 those regulate the expression of miR-22^5,13,16,20,26,27^. In addition, HIP/RPL29 expression is regulated by both sodium butyrate and glucose deprivation in HT-29 cells and results in induction of cellular differentiation^42^. These findings are supportive of our results. So far there are no reports on the existence of the common factor, which can similarly regulate expression of both molecules RPL29 and miR22. On the other hand, there is more evidence suggesting the connection between RLP29 and c-Fos. Heparin is a potent inhibitor of c-Fos, therefore, RPL29 may bind to heparin and induce c-Fos expression^43^. C-Fos is a predicted transcription factor of FosB, however, there is no direct evidence yet. Further studies are needed to find out the mechanism of c-Fos and FosB regulation by RPL29.

In addition, RPL29 can amplify the rate of protein synthesis by enhancing efficiency of the ribosomal translational machinery and plays a critical roles in diverse biological processes such as cell cycle control, cell proliferation, cell differentiation and angiogenesis^29,30^. Its deficiency impairs embryonic development and consequently results in growth retardation of mouse^44,45^. It regulates differentiation and growth of chondrocytes and enhances the amplification of proteins that are critical for controlling cell cycle during skeletal development and useful markers for the development of normal bone mass and quality^30,45^. It also induces cell proliferation in the colon and pancreatic cancer^42,46^. The depletion of RPL29 results in suppression of cell proliferation, induced cell cycle arrest at G0/G1 phase and enhanced cell apoptosis in pancreatic cancer cell^46^. In addition, knockdown of this gene stimulates cell differentiation accompanied by the upregulation of two potential cell differentiation markers mucin-2 and galectin-4 in colon cancer cells^42^. Endogenous RPL29 and miR22 can also regulate angiogenesis. Some scientists reported that tumor angiogenesis is enhanced in mice lacking β3-integrins whereas RPL29 is significantly upregulated in β3-null endothelial cells and regulates angiogenesis via controlling VEGF^47^. Therefore, RPL29 is a potential factor of angiogenesis and the depletion, or loss of this protein can reduce angiogenesis^47,48^. Deficiency of this gene invariably associated with a flagellar morphological anomaly of mammalian sperm termed as ‘dag’ and consequently results in infertility along with low sperm motility^49^. RPL29 also regulates depression and anxiety-like behavior^50^. However, there are no references about the regulatory role of RPL29 on miR22 expression. For the first time, we identified that the different expression of RPL29 might be a reason for tissue-specific expression of miR22. It needs further studies to determine the pathways in details about the regulation of miR22 by RPL29. We believe that our findings would be helpful to find out way of controlling miR22 depending on the cells or tissues in different diseased conditions.

## Materials and methods

### Cell culture and treatment

Human glioblastoma astrocytoma cells (U251), and human umbilical vein cells (EA.hy926 cell line, fusion of endothelial cell—primary human umbilical vein cells with the carcinomatous lung epithelial cell A549 by exposure to polyethylene glycol) were cultured in DMEM (Cat. #SH3002201, HyClone, Logan, UT, USA) medium and maintained in humidified air at 37 °C with 5% CO_2_. The medium was supplemented with 10% fetal bovine serum (Cat. #1027-106, Gibco, Walthan, MA, USA), 100 mg/ml streptomycin and 100 U/ml penicillin (Invitrogen, Carlsbad, CA, USA). Cells were plated in 12-well plates (1 × 10^5^) and transfected siRNAs using Lipofectamine-2000 (Invitrogen) at the 70–80% of confluence level. Twenty-four hours later the cells were suggested to poly(I:C) treatment for the indicated period. EA.hy926 cells were obtained from ATCC (Manassas, VA, USA) and U251 cells were the kind gift from Professor Shengbo Cao, College of Veterinary Medicine, Huazhong Agricultural University.

### Animal experiment

C57BL/6 mice (7 weeks old, male) were obtained from the Laboratory Animal Center of Huazhong Agricultural University, Wuhan, China. Mice were reared about 1 week for adaptation and randomly divide into control and treatment group (n = 9). Poly(I:C) potassium salt (Sigma-Aldrich, St. Louis, MO, USA) was diluted in normal saline (NS) at the concentration of 1 µg/µl. Mice were then treated with 100 µl NS and 100 µg of poly(I:C)/mouse respectively through intraperitoneal injection for 24 h. The mice were sacrificed after treatment period to collect brain and lung tissues following institutional guideline. The collected tissues of both the control and treated mice were subjected to RNA extraction.

### RNA extraction and quantitative real-time PCR

Total RNA was extracted from mouse lung and brain tissues and from cells using the reagent RNA-iso Plus (Takara Bio Inc. Kusatsu, Shiga, Japan) as per the manufacturer’s instruction. The RNA concentration was assessed using NanoDrop 2000 (Thermo Fisher Scientific, Wilmington, DE19810, USA). Reverse transcription was performed using 1 µg total RNA as the template with PrimeScript-RT reagent Kit with gDNA Eraser (Cat. #RR047A, Takara) using oligodT, miR22 and U6 specific RT-primers according to the kits instruction. Quantitative real-time PCR (qPCR) was performed for quantification of genes expression using LightCycler-96 qPCR detection system (Roche) and SYBR Premix Ex Taq II (Cat. #RR820A, Takara) according to the manufacturer’s protocol. The information about primers used for qRT-PCR has enlisted in Table 1. The gene’s expression has standardized to that of the control sample, GAPDH and U6 were used as internal control, and fold change was calculated using the 2^−ΔΔCt^ method. The thermal cycles of qRT-PCR were 95 °C for 300 s followed by 40 cycles (95 °C for 15 s, 58^◦^C for 30 s) and 72 °C for 30 s.

**Table 1:** List of primers used in qRT-PCR.

Species	Gene	Forward sequence 5′- > 3 ‘	Reverse sequence 5′- > 3 ‘
Mouse	Rpl29	GATGCAGGCCAACAATGCAA	CTTAGGCTTCGGTTGGCAGA
	Gapdh	AAATGGTGAAGGTCGGTGTGAAC	TGAAGGGGTCGTTGATGGC
Human	GAPDH	AACGGATTTGGTCGTATTGGG	CCTGGAAGATGGTGATGGGAT
	RPL29	GGCGTTGTTGACCCTATTTC	TGTGTGGTGTGGTTCTTGGA
	PU.1	GAAGGACAGCATCTGGTGGGT	GCCGTCTTGCCGTAGTTGC
	Fos-B	GACCCCGAGAGGAGACGCTCA	CAACTGATCTGTCTCCGCCTGG
	c-Fos	CAGACTACGAGGCGTCATCC	TCTGCGGGTGAGTGGTAGTA
Human and mouse	U6	CTCGCTTCGGCAGCACA	AACGCTTCACGAATTTGCGT
	miR22	AAGCUGCCGUUGAAGAACUGU	GTGCAGGGTCCGAGGT
	U6 RT primer	GTCGTATCCAGTGCAGGGTCCGAGGTGCACTGGATACGACAAAATATGG	
	miR22 RT primer	GTCGTATCCAGTGCAGGGTCCGAGGTATTCGCACTGGATACGACACAGTT	

### Western blotting

The mouse brain and lung tissues were lysed in RIPA buffer (Cat. #PP1202, Aidlab Biotechnologies, Beijing, China) containing 150 mM NaCl, 0.1% Triton X-100, 0.5% sodium deoxycholate, 0.1% SDS (Sodium dodecyl sulphate), 50 mM Tris–HCl, pH 8.0, Protease inhibitors. The tissue lysates were centrifuged for 20 min at 12,000*g* and 4 °C and total protein was collected as supernatant. The concentration of the total protein was assayed using bicinchoninic acid method (Cat. #PP0101, Aidlab Biotechnologies). The proteins were separated using SDS-PAGE gel and transferred to PVDF membrane. After transfer, the PVDF membrane was cut down into different pieces according the sizes of the RPL-29 and GAPDH with the guidance of molecular weight markers. The membranes were incubated for 2 h at room temperature in blocking buffer (20 mM Tris–HCl, 137 mM NaCl, pH 8.0, containing 0.1% Tween and 5% non-fat dry milk). After blocking, the membranes were probed with antibodies against RPL-29 (1:1000, Cat. # 15799-1-AP, Proteintech, Wuhan, China) and GAPDH (1:5000, GB12002, Servicebio, Wuhan, China) overnight at 4 °C. The positive signals for proteins were detected utilizing ECL reagents (BIO-RAD, Hercules, CA, USA) and image capturing system (Biotanon, Shanghai, China) after probing with secondary antibody for 2 h at room temperature. The proteins ratio was determined using software “Image J” densitometric analysis.

### Statistical analysis

Each experiment was performed a minimum three times having similar results. We used the GraphPad Software Prism-6 (San Diego, CA, USA) to analyze the qRT-PCR results and represented the values as mean ± standard deviation. Data were compared with two-tailed unpaired Student's t test. For all tests, we considered the differences as significant with the p < 0.05.

### Ethics statement

Animal handling and experimental procedures were approved by the Animal Care and Use Committee of Hubei Province, China and executed in accordance with guidelines developed by the China Council on Animal Care and Protocol. The ethics approval number was HZAHMD-2016-037.

## Supplementary information

Supplementary Information 1

## References

[1] Ambros V (2004). The functions of animal microRNAs. Nature.

[2] Bartel DP (2004). MicroRNAs: Genomics, biogenesis, mechanism, and function. Cell.

[3] Rodriguez A, Griffiths-Jones S, Ashurst JL, Bradley A (2004). Identification of mammalian microRNA host genes and transcription units. Genome Res..

[4] Jovicic A, Jolissaint JFZ, Moser R, Santos MDFS, Luthi-Carter R (2013). MicroRNA-22 (miR-22) overexpression is neuroprotective via general anti-apoptotic effects and may also target specific Huntington’s disease-related mechanisms. PLoS One.

[5] Gu W (2017). MicroRNA-22 regulates inflammation and angiogenesis via targeting VE-cadherin. FEBS Lett..

[6] Xu D (2011). miR-22 represses cancer progression by inducing cellular senescence. J. Cell. Biol..

[7] Xin M (2016). miR-22 inhibits tumor growth and metastasis by targeting ATP citrate lyase: Evidence in osteosarcoma, prostate cancer, cervical cancer and lung cancer. Oncotarget.

[8] Hong Y (2016). MiR-22 may suppress fibrogenesis by targeting TGFβR I in cardiac fibroblasts. Cell Physiol. Biochem..

[9] Tang Y (2015). microRNA-22 acts as a metastasis suppressor by targeting metadherin in gastric cancer. Mol. Med. Rep..

[10] Lu W (2015). The microRNA miR-22 inhibits the histone deacetylase HDAC4 to promote T H 17 cell–dependent emphysema. Nat. Immunol..

[11] Iliopoulos D, Malizos KN, Oikonomou P, Tsezou A (2008). Integrative microRNA and proteomic approaches identify novel osteoarthritis genes and their collaborative metabolic and inflammatory networks. PLoS One.

[12] Li HS, Greeley N, Sugimoto N, Liu YJ, Watowich SS (2012). miR-22 controls Irf8 mRNA abundance and murine dendritic cell development. PLoS One.

[13] Ming G-F, Wu K, Hu K, Chen Y, Xiao J (2016). NAMPT regulates senescence, proliferation, and migration of endothelial progenitor cells through the SIRT1 AS lncRNA/miR-22/SIRT1 pathway. Biochem. Biophys. Res. Commun..

[14] Yang J (2016). microRNA-22 attenuates myocardial ischemia-reperfusion injury via an anti-inflammatory mechanism in rats. Exp. Ther. Med..

[15] Lin J (2014). A novel p53/microRNA-22/Cyr61 axis in synovial cells regulates inflammation in rheumatoid arthritis. Arthritis Rheumatol..

[16] Jiang R (2011). miR-22 promotes HBV-related hepatocellular carcinoma development in males. Clin. Cancer Res..

[17] Ohnishi Y-I (2016). Promotion of astrocytoma cell invasion by micro RNA–22 targeting of tissue inhibitor of matrix metalloproteinase–2. J. Neurosurg. Spine.

[18] Wang J (2017). Molecular mechanisms and clinical applications of miR-22 in regulating malignant progression in human cancer (Review). Int. J. Oncol..

[19] Gupta SK (2016). Preclinical development of a microRNA-based therapy for elderly patients with myocardial infarction. J. Am. Coll. Cardiol..

[20] He J (2016). Influence and significance of intervening diabetes microRNA expression profile of NOD mice with exendin-4. Eur. Rev. Med. Pharmacol. Sci..

[21] Yang CP, Zhang ZH, Zhang LH, Rui HC (2016). Neuroprotective role of microRNA-22 in a 6-hydroxydopamine-induced cell model of Parkinson’s disease via regulation of its target gene TRPM7. J. Mol. Neurosci..

[22] Huang ZP, Wang DZ (2014). miR-22 in cardiac remodeling and disease. Trends Cardiovasc. Med..

[23] Yamakuchi M, Yagi S, Ito T, Lowenstein CJ (2011). MicroRNA-22 regulates hypoxia signaling in colon cancer cells. PLoS One.

[24] Akamatsu S (2015). Differences in serum microRNA profiles in hepatitis B and C virus infection. J. Infect..

[25] Shi C, Xu X (2013). MicroRNA-22 is down-regulated in hepatitis B virus-related hepatocellular carcinoma. Biomed. Pharmacother..

[26] Xu D, Guo Y, Liu T, Li S, Sun Y (2017). miR-22 contributes to endosulfan-induced endothelial dysfunction by targeting SRF in HUVECs. Toxicol. Lett..

[27] Ahmad HM, Muiwo P, Muthuswami R, Bhattacharya A (2017). FosB regulates expression of miR-22 during PMA induced differentiation of K562 cells to megakaryocytes. Biochimie.

[28] Wan S (2016). MicroRNA-22 negatively regulates poly (I: C)-triggered type I interferon and inflammatory cytokine production via targeting mitochondrial antiviral signaling protein (MAVS). Oncotarget.

[29] Hamidi T (2018). Identification of Rpl29 as a major substrate of the lysine methyltransferase Set7/9. J. Biol. Chem..

[30] Oristian DS (2010). Ribosomal protein L29/HIP deficiency delays osteogenesis and increases fragility of adult bone in mice. J. Orthop. Res..

[31] Ali M (2019). Differential expression of toll-like receptor 13 and ribosomal protein L29 in inflammatory lung and brain. J. Biol. Regul. Homeost. Agents.

[32] Lee JH (2017). c-Fos-dependent miR-22 targets MDC1 and regulates DNA repair in terminally differentiated cells. Oncotarget.

[33] Shen C (2016). The PU.1-modulated microRNA-22 Is a regulator of monocyte/macrophage differentiation and acute myeloid leukemia. PLoS Genet..

[34] Bar N, Dikstein R (2010). miR-22 forms a regulatory loop in PTEN/AKT pathway and modulates signaling kinetics. PLoS One.

[35] Li J (2015). Difference in microRNA expression and editing profile of lung tissues from different pig breeds related to immune responses to HP-PRRSV. Sci. Rep..

[36] Moheimani F (2018). Influenza A virus infection dysregulates the expression of microRNA-22 and its targets; CD147 and HDAC4, in epithelium of asthmatics. Respir. Res..

[37] Ma X (2018). Differentially expressed non-coding RNAs induced by transmissible gastroenteritis virus potentially regulate inflammation and NF-κB pathway in porcine intestinal epithelial cell line. BMC Genom..

[38] Xiao S (2016). MiR-22 promotes porcine reproductive and respiratory syndrome virus replication by targeting the host factor HO-1. Vet. Microbiol..

[39] Gidlöf O (2015). Extracellular uridine triphosphate and adenosine triphosphate attenuate endothelial inflammation through miR-22-mediated ICAM-1 inhibition. J. Vasc. Res..

[40] Chen B (2016). miR-22 contributes to the pathogenesis of patients with coronary artery disease by targeting MCP-1: An observational study. Medicine.

[41] Sibbesen NA (2015). Jak3, STAT3, and STAT5 inhibit expression of miR-22, a novel tumor suppressor microRNA, in cutaneous T-cell lymphoma. Oncotarget.

[42] Jian-Jun L, Hua HB, Jinqiu Z, Carson DD, Shing Chuan H (2010). Repression of HIP/RPL29 expression induces differentiation in colon cancer cells. J. Cell. Physiol..

[43] Pukac L, Castellot J, Wright T, Caleb B, Karnovsky M (1990). Heparin inhibits c-fos and c-myc mRNA expression in vascular smooth muscle cells. Cell Regul..

[44] Kirn-Safran CB (2007). Global growth deficiencies in mice lacking the ribosomal protein HIP/RPL29. Dev. Dyn..

[45] Miller SA, Brown AJ, Farach-Carson MC, Kirn-Safran CB (2010). HIP/RPL29 down-regulation accompanies terminal chondrocyte differentiation. Differentiation.

[46] Li C, Ge M, Yin Y, Luo M, Chen D (2012). Silencing expression of ribosomal protein L26 and L29 by RNA interfering inhibits proliferation of human pancreatic cancer PANC-1 cells. Mol. Cell Biochem..

[47] Jones DT (2013). Endogenous ribosomal protein L29 (RPL29): A newly identified regulator of angiogenesis in mice. Dis. Model. Mech..

[48] Sonia DS (2010). HIP/RPL29 antagonizes VEGF and FGF2 stimulated angiogenesis by interfering with HS-dependent responses. J. Cell Biochem..

[49] Rolands (2014). Ultrastructural changes and asthenozoospermia in murine spermatozoa lacking the ribosomal protein L29/HIP gene. Asian J. Androl..

[50] Smagin DA (2015). Dysfunction in ribosomal gene expression in the hypothalamus and hippocampus following chronic social defeat stress in male mice as revealed by RNA-Seq. Neural. Plast..

