# Supplementary material for: The tissue specific regulation of miR22 expression in the lung and brain by ribosomal protein L29
Source: Sci Rep. 2020 Oct 1;10:16242. doi: 10.1038/s41598-020-73281-z (PMC7530758; doi:10.1038/s41598-020-73281-z)
Supplement: Supplementary file 1 — Supplementary Information 1 [file 41598_2020_73281_MOESM1_ESM.pdf]

The Tissue Specific Regulation of miR22 Expression in the Lung and Brain by Ribosomal Protein L29

Mohammad Ishaque Ali [2, 3], Linrui Li[2], Lexing Li[2], Lun Yao[2], Jie Liu[2], Wei Gu[1], Shuguang Huang[2], Bingyu Wang[2], Guoquan Liu[1, 2*]

[1] Department of Biochemistry and Molecular Biology, School of Medical Laboratory, and Anhui Province Key Laboratory of Translational Cancer Research, Bengbu Medical College, Bengbu, Anhui Province 233030, People's Republic of China.

[2] Department of Basic Veterinary Medicine, College of Veterinary Medicine, Huazhong Agricultural University, Wuhan, Hubei Province 430070, People's Republic of China.

[3] Department of Livestock Services, Dhaka, People's Republic of Bangladesh.

*Corresponding Author:

Guoquan Liu

Department of Biochemistry and Molecular Biology, School of Laboratory Medicine, Bengbu Medical College

2600 Donghai Street, Bengbu, Anhui Province, People's Republic of China 233030

E-mail: guoquanliu@bbmc.edu.cn

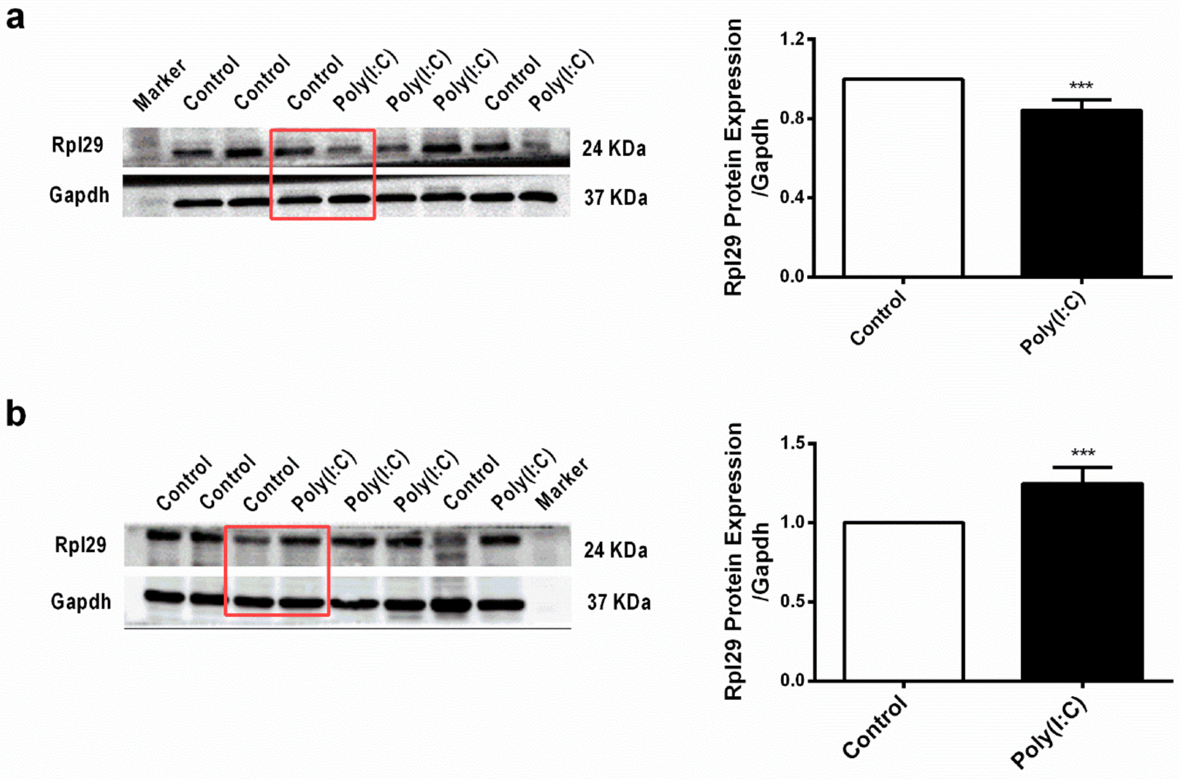

Supplementary Figure S1: RPL29 protein expression in the brain and lung tissues of mouse in response to poly(I:C). Mice were treated with 100 µg of poly(I:C) for 24 hours and RPL29 protein was quantified in the lung (**a**) and brain (**b**) tissues of through western blot. The fisrt part of both the images of **a** and **b** are croped from full-length blots/gels are presented in Supplementary Figure S2-S5. The region of a and b marked by the red box are included in the figure 2b and 2d respectively.

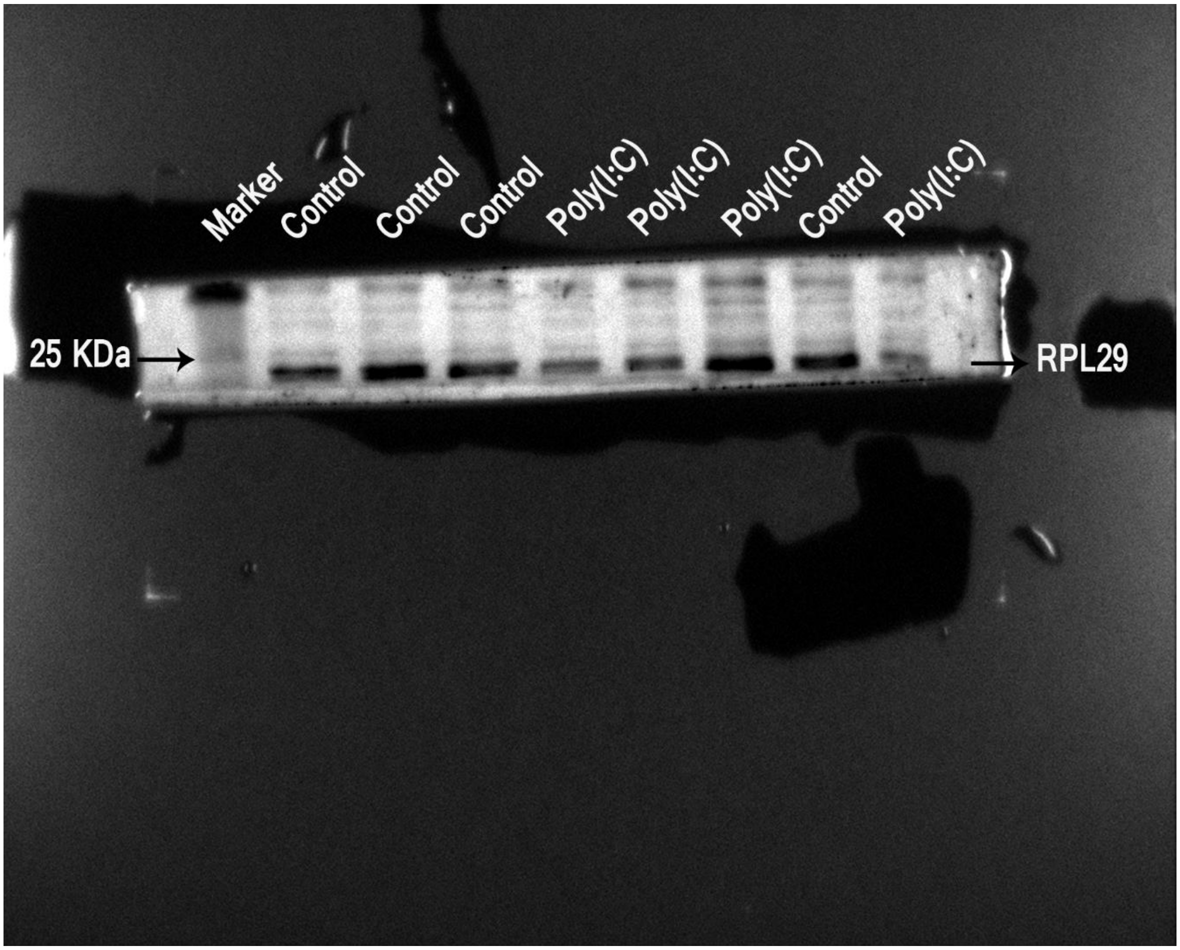

Supplementary Figure S2: Full-length blots/gel of Rpl29 in lung tissues of poly(I:C) treated mouse.

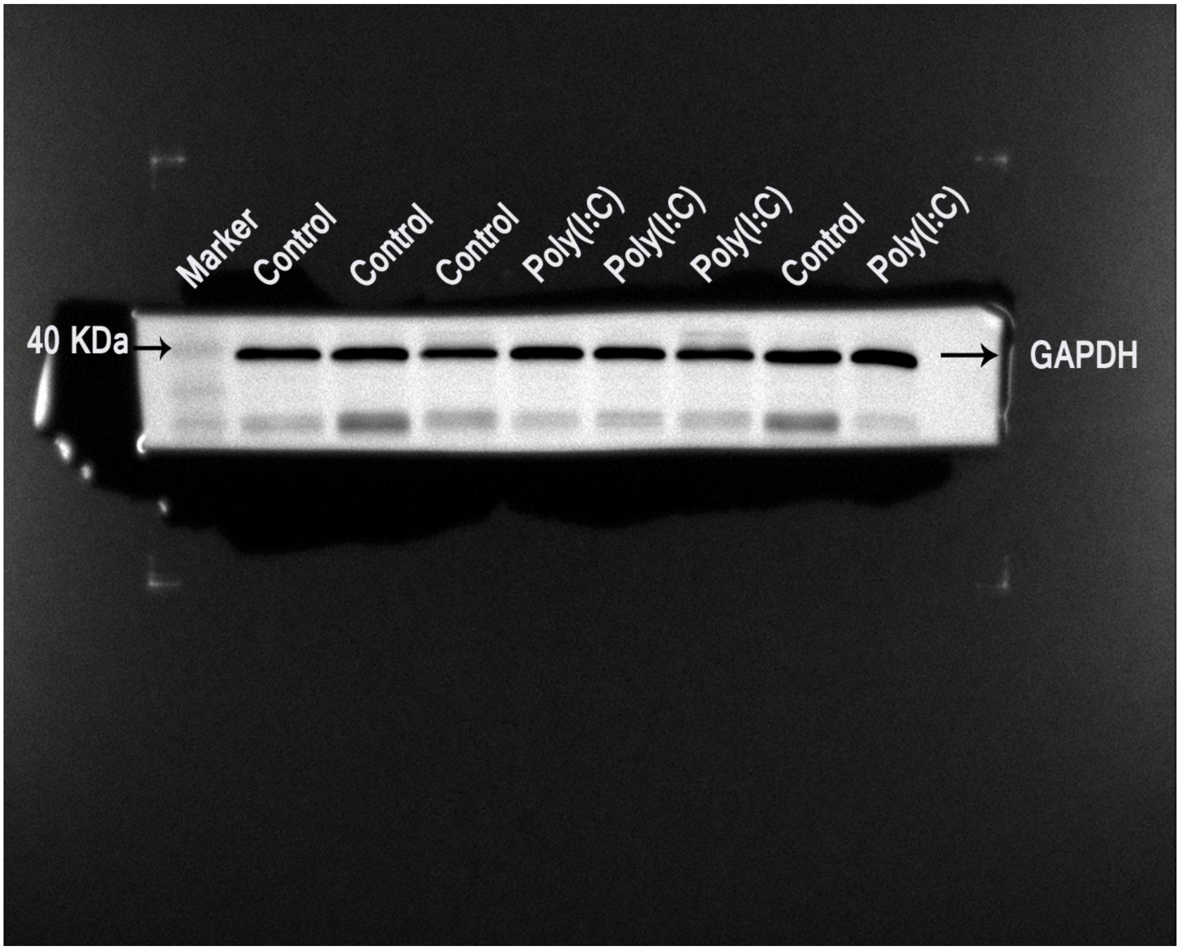

Supplementary Figure S3: Full-length blots/gel of Gapdh in lung tissues of poly(I:C) treated mouse.

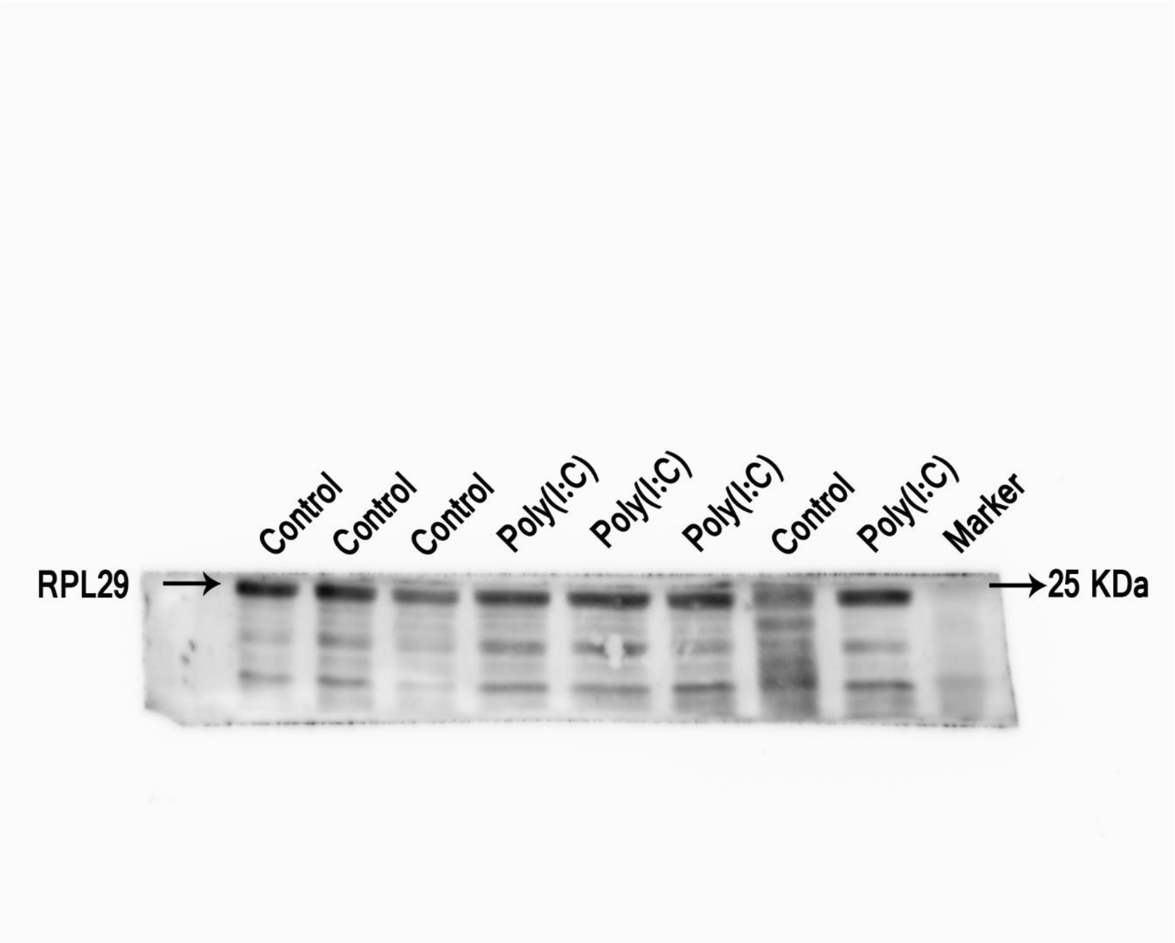

Supplementary Figure S4: Full-length blots/gel of Rpl29 in brain tissues of poly(I:C) treated mouse.

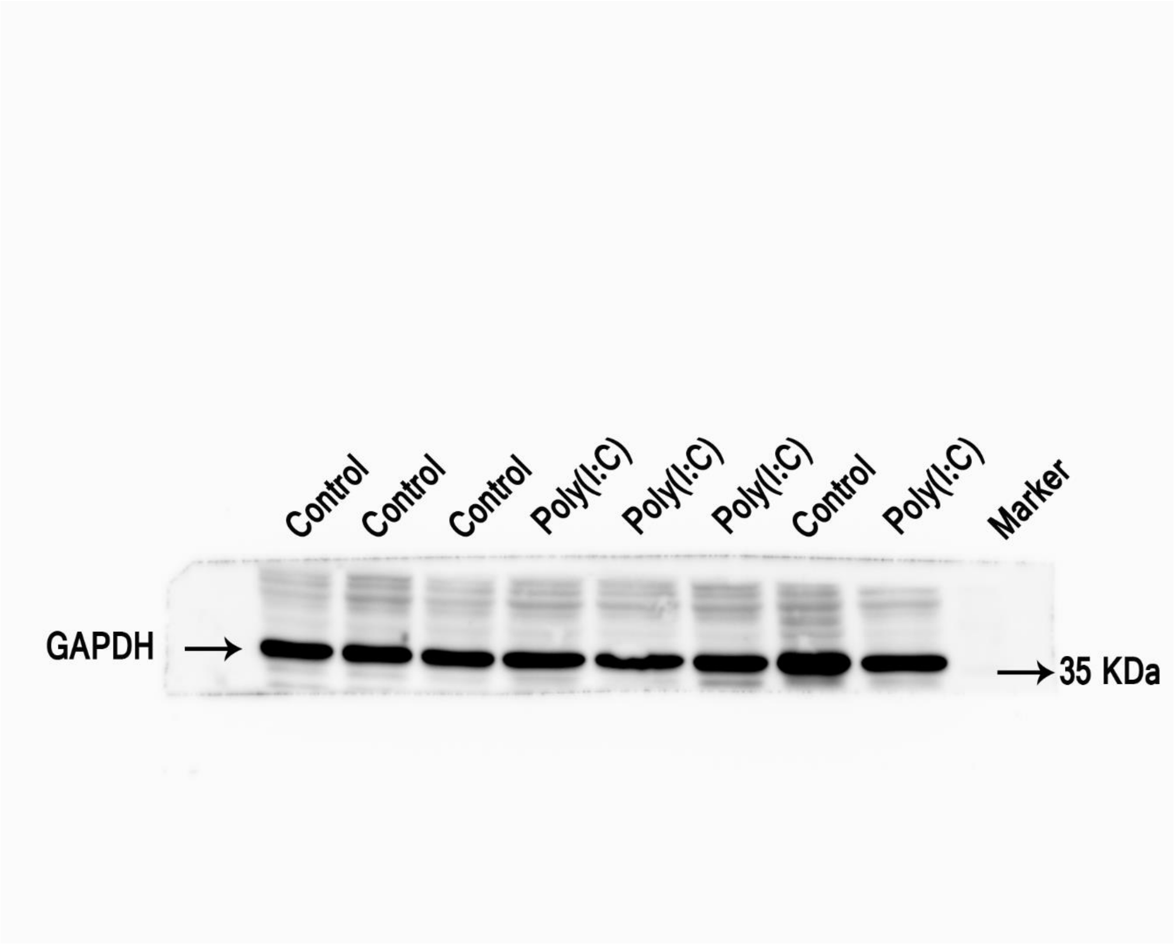

Supplementary Figure S5: Full-length blots/gel of Gapdh in brain tissues of poly(I:C) treated mouse.
